# Hereditary Erythema

**Published:** 1865-11

**Authors:** C. A. White

**Affiliations:** Iowa City, Iowa


### HEREDITARY ERYTHEMA.

**Reported by C. A. WHITE, M. D., of Iowa City, Iowa.**

A few months since I was called to vaccinate a man of 36 years, a native of Wales, together with his son, a lad of 8 years, both of whom, I noticed, had very red faces. The father informed me that they had both been so from their birth. The redness resembled in appearance what we often see in summer in cases of persons having exposed to the sun for a short time, parts of the body which are usually covered. It would disappear under pressure, and immediately reappear when the pressure was removed. Both have always enjoyed the most robust health, and have never felt pain in the parts thus affected.

It covered the whole face *symmetrically*, extending from just beneath the chin to the lower portion of each temple, thence forming an obtuse angle on the forehead. This outline was identical in both father and son. The skin had no appearance of tumefaction nor disease.

